# Leveraging Acquired EGFR-TKI-Resistant Models to Identify MUC16 as a Therapeutic Vulnerability in Lung Adenocarcinoma

**DOI:** 10.3390/ph19010047

**Published:** 2025-12-25

**Authors:** Yinhua Tan, Chunxiu Xiao, Zhifan Wang, Yuhang Kong, Yamei Huang, Zhichang Liu, Qiang Wu, Chenyu Wu, Manyu Zhao, Jingyao Chen, Kai Xiao

**Affiliations:** 1 Laboratory of Precision Therapeutics, Department of Pulmonary and Critical Care Medicine, State Key Laboratory of Respiratory Health and Multimorbidity, Frontiers Science Center for Disease-Related Molecular Network, West China Hospital, Sichuan University, Chengdu 610041, China; 2 Frontier Medical Center, Tianfu Jincheng Laboratory, Chengdu 610041, China

**Keywords:** lung adenocarcinoma, EGFR-TKIs, *MUC16*, drug resistance, transcriptomics

## Abstract

**Background/Objectives**: Acquired resistance to epidermal growth factor receptor (EGFR)-tyrosine kinase inhibitors (TKIs) remains a major challenge in the treatment of EGFR-mutant lung adenocarcinoma (LUAD). This study aimed to develop and characterize representative models of acquired EGFR-TKI resistance and to identify potential therapeutic targets mediating this process. **Methods**: Resistant models of PC9 and LUAD-PDCs were generated using a standardized dose-escalation protocol. The resulting models were characterized by drug response assays, morphology, and transcriptomic sequencing. Candidate target genes were validated across all resistant models using siRNA knockdown followed by re-sensitization assays. Clinical relevance was further examined through analysis of publicly available datasets. **Results**: These generated models displayed stable resistant phenotypes and unique transcriptomic alterations. Cross-model analysis revealed *MUC16* as a consistently upregulated gene associated with resistance. Functional validation demonstrated that *MUC16* depletion re-sensitized all resistant models to EGFR-TKIs. Furthermore, analysis of clinical data linked high *MUC16* expression to poorer patient outcomes. **Conclusions**: This study establishes stable in vitro models for investigating acquired resistance in EGFR-mutant LUAD and identifies *MUC16* as a functionally validated and clinically relevant mediator of EGFR-TKI resistance, providing a potential therapeutic target for overcoming drug resistance.

## 1. Introduction

Lung cancer remains the most common cause of cancer-related mortality globally, responsible for an estimated 1.8 million deaths annually. Among its subtypes, lung adenocarcinoma (LUAD) represents the predominant histology, comprising approximately 40–50% of all cases [1,2]. The epidermal growth factor receptor (*EGFR*) gene is the most frequently mutated driver in LUAD, with sensitive mutations occurring in about 50% of patients in East Asian populations [3,4]. In recent decades, the development and clinical application of EGFR tyrosine kinase inhibitors (EGFR-TKIs), from first- and second-generation agents such as Gefitinib and Erlotinib to third-generation drugs like Osimertinib, have substantially transformed the therapeutic landscape for this disease [5]. Nevertheless, despite marked initial responses, acquired resistance inevitably emerges in the vast majority of patients, typically after 9–18 months of treatment [6,7]. Consequently, overcoming EGFR-TKI resistance represents one of the most pressing challenges in the management of LUAD.

The stepwise dose-escalation of EGFR-TKIs serves as a well-established approach for generating in vitro resistance models that faithfully recapitulate adaptive processes under therapeutic pressure. Previous studies utilizing this methodology have revealed diverse resistance mechanisms: chronic Gefitinib exposure in PC9 cells induced T790M mutation [8], repeated treatment in A549 cells triggered epithelial–mesenchymal transition (EMT) [9], and prolonged selection in PC9 cells uncovered altered adaptor-protein signaling [10]. Building on this foundation, a similar dose-escalation protocol was implemented to develop EGFR-TKI-resistant variants from both PC9 cells and LUAD-PDCs. This complementary model system integrates the reproducibility of established cell lines with the clinical relevance of PDCs, creating a robust platform for investigating EGFR-TKIs resistance mechanisms.

The mechanisms underlying acquired resistance to EGFR-TKIs are multifactorial and complex, encompassing on-target secondary EGFR mutations (e.g., T790M and C797S), off-target activation of bypass signaling pathways (such as *MET* amplification, *HER2* alterations, and *IGF1R* activation), phenotypic transformation such as EMT, and remodeling of the tumor microenvironment [6,11,12]. While these established mechanisms explain a proportion of resistance cases, a significant number of patients develop resistance through elusive or non-canonical pathways [13,14,15]. Therefore, systematic identification and functional validation of genes capable of reversing or modulating the resistant phenotype, beyond currently known mechanisms, are essential to expand the repertoire of druggable targets and provide additional avenues for overcoming EGFR-TKI resistance.

To address these challenges, this study established a comprehensive panel of EGFR-TKI-resistant models using PC9 cells and PDCs. This platform serves as a robust tool for the systematic identification and validation of resistance mediators. Furthermore, based on these resistant models, transcriptomic analyses and functional assays identified *MUC16* as a potential therapeutic target for overcoming drug resistance.

## 2. Results

### 2.1. Validation of Baseline EGFR-TKI Sensitivity

#### 2.1.1. In Vitro EGFR-TKI Sensitivity

**Figure 1:**
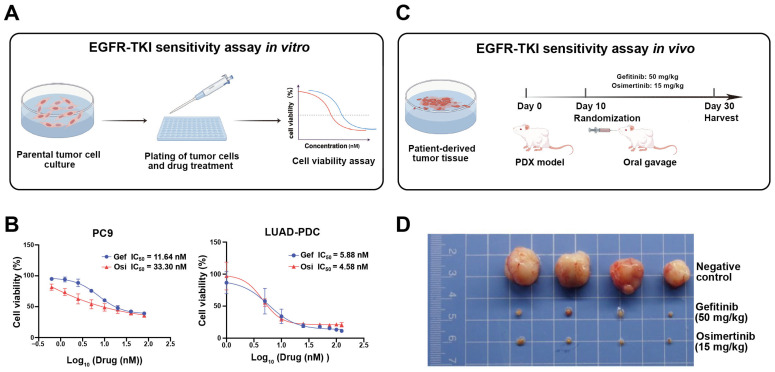
Validation of baseline EGFR-TKI sensitivity in parental PC9 and LUAD-PDC cells. (**A**) Schematic diagram of the in vitro drug sensitivity assay using CCK-8. Parental cells were treated with gradient concentrations of Gefitinib (blue) or Osimertinib (red) for 48 h, followed by cell viability measurement. (**B**) Dose–response curves of parental PC9 and LUAD-PDC cells treated with Gefitinib or Osimertinib for 48 h. (**C**) Schematic illustration of the in vivo subcutaneous xenograft model. Mice bearing LUAD-PDC-derived tumors were randomized into control, Gefitinib, or Osimertinib treatment groups. (**D**) Representative images of excised tumors from each treatment group at the endpoint of the in vivo study.

We first rigorously characterized the intrinsic EGFR-TKI sensitivity of the parental PC9 and LUAD-PDC cells, which both carry EGFR exon 19 deletion mutations [16,17] (Figure 1A). CCK-8 cell viability assays confirmed that both cell lines were highly susceptible to EGFR inhibition. Quantitatively, PC9 cells exhibited IC_50_ values of 11.64 nM for Gefitinib and 33.30 nM for Osimertinib, while the LUAD-PDCs showed IC_50_ values of 5.88 nM and 4.58 nM, respectively (Figure 1B).

#### 2.1.2. In Vivo EGFR-TKI Sensitivity

To further validate the sensitivity of LUAD-PDC cells in vivo, subcutaneous xenograft models were established in immunodeficient mice. Animals were randomized into three groups: Gefitinib-treated, Osimertinib-treated, and vehicle control (Figure 1C). Both EGFR-TKI treatments significantly suppressed tumor growth compared to the control group, consistent with the in vitro sensitivity profiles (Figure 1D).

### 2.2. Generation of Stable EGFR-TKI-Resistant Models

**Figure 2:**
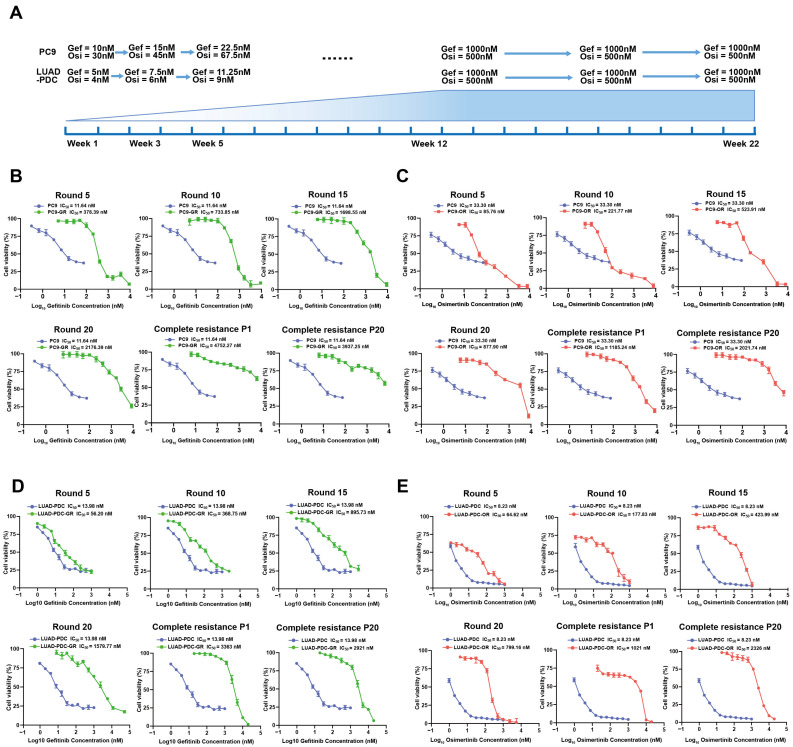
Establishment and validation of EGFR-TKI-resistant models induced from PC9 and LUAD-PDC. (**A**) Schematic illustration of the prolonged dose-escalation protocol for generating resistant variants. PC9 and LUAD-PDC were exposed to incrementally increasing concentrations of Gefitinib or Osimertinib over 12 weeks, followed by maintenance at stable therapeutic doses until week 22. (**B**–**E**) Dose–response curves for PC9 cells during the development of resistance to (**B**) Gefitinib and (**C**) Osimertinib, and for LUAD-PDC cells during resistance to (**D**) Gefitinib and (**E**) Osimertinib, showing the progression from parental to fully resistant states.

We established EGFR-TKI-resistant models using a dose-escalation protocol adapted from established methods [8,16]. Both PC9 and LUAD-PDC cells were continuously exposed to incrementally increasing concentrations of Gefitinib or Osimertinib for 22 weeks to generate resistant variants (Figure 2A).

To monitor the acquisition of resistance, IC_50_ values were measured at regular intervals during the induction process. This revealed a progressive and substantial increase in drug tolerance for both cell lines and both inhibitors (Figure 2B–E). Functional validation of the fully resistant cells confirmed the development of stable, high-level resistance. In PC9 cells, the IC_50_ values for Gefitinib- and Osimertinib-resistant (PC9-GR, PC9-OR) cells increased by >300-fold and >60-fold, respectively, compared to parental cells. Similarly, the LUAD-PDC-derived resistant models (LUAD-PDC-GR, LUAD-PDC-OR) exhibited >200-fold and >250-fold increases in IC_50_ values, respectively (Figure 2B–E). These results collectively demonstrate the successful establishment of stable and robust EGFR-TKI-resistant models using this standardized protocol.

### 2.3. Phenotypic and Molecular Characterization of EGFR-TKI-Resistant LUAD-PDC Models

#### 2.3.1. Resistant Models Maintain Morphological Integrity and Lineage Markers

**Figure 3:**
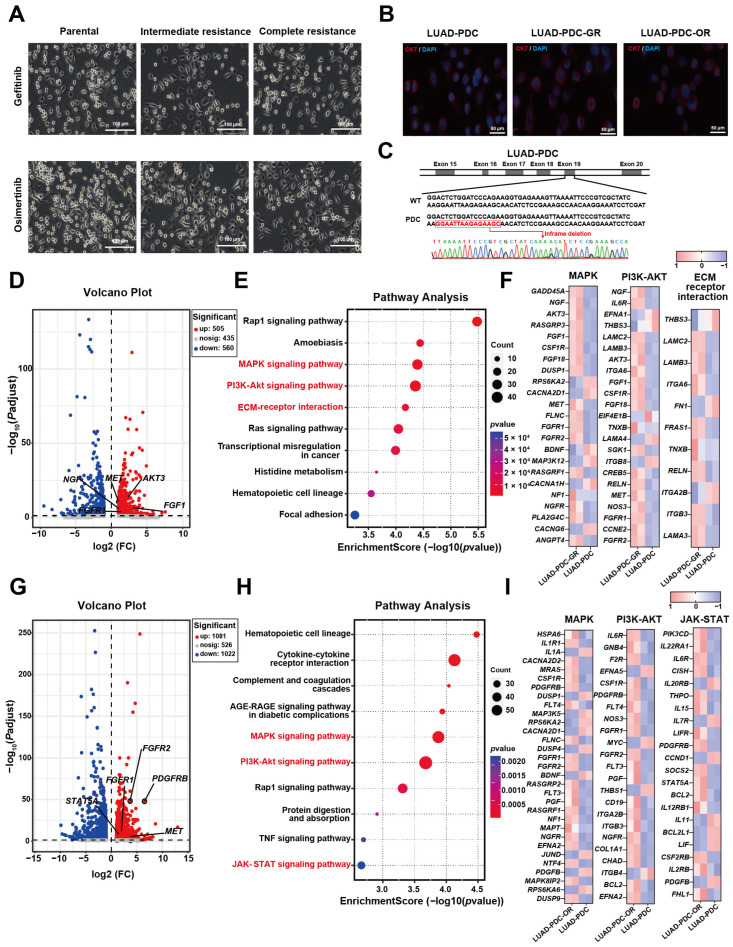
Phenotypic and molecular characterization of resistant LUAD-PDC models. (**A**) Representative images comparing the morphology of parental, intermediate, and complete resistant LUAD-PDC cells. (**B**) Immunofluorescence staining of CK7 in parental and resistant LUAD-PDC cells. (**C**) The parental LUAD-PDC had an exon 19 deletion mutation. (**D**–**F**) Transcriptomic analysis of Gefitinib-resistant model: (**D**) Volcano plot of differentially expressed genes in LUAD-PDC-GR versus parental cells. (**E**) KEGG pathway enrichment analysis. (**F**) Heatmap displaying expression patterns of key genes from the pathways enriched in (**E**). (**G**–**I**) Transcriptomic analysis of Osimertinib-resistant model: (**G**) Volcano plot of differentially expressed genes in LUAD-PDC-OR versus parental cells. (**H**) KEGG pathway enrichment analysis. (**I**) Heatmap of key gene expression from the pathways in (**H**).

Both LUAD-PDC-GR and LUAD-PDC-OR cells maintained characteristic spindle-shaped morphologies from the sensitive parental stage through intermediate resistance to fully resistant states (Figure 3A). These data indicate that the cellular architecture remains epithelial despite progressive resistance to EGFR-TKI, and no overt phenotypic transition was observed during this process.

To molecularly characterize the resistant phenotypes, the LUAD lineage was first confirmed using cytokeratin 7 (CK7). This marker was selected based on its integral role in the pathological distinction between LUAD and squamous cell carcinoma (LUSC). CK7 is a cornerstone of the standard diagnostic IHC panel, and its expression supports LUAD diagnosis, while CK5/6 is definitive for LUSC [18]. Immunofluorescence analysis revealed that CK7 expression was maintained in LUAD-PDC-GR and LUAD-PDC-OR cells compared to their corresponding parental controls (Figure 3B). The persistent expression of this epithelial differentiation marker over multiple passages confirms the maintenance of adenocarcinoma identity during the development of drug resistance.

The genetic basis of the models was further characterized through Sanger sequencing of EGFR in parental LUAD-PDC cells, which confirmed a canonical exon 19 deletion (Figure 3C), consistent with their initial sensitivity to EGFR-TKIs. Additionally, short tandem repeat (STR) profiling demonstrated a high-degree match between the parental and resistant variants (Table 1, Table 2 and Table 3), confirming their common genetic origin and lineage stability throughout the resistance induction process.

**Table 1:** Assessment of STR genetic markers between samples.

Sample	Sample	EV Score	Degree of Match
LUAD-PDC	LUAD-PDC-GR	1.00	100%
LUAD-PDC	LUAD-PDC-OR	1.00	100%

Notes: EV score = (2 × NO. shared alleles)/(NO. query alleles + NO. reference alleles).

**Table 2:** Cellular STR loci and genotyping results in LUAD-PDC and LUAD-PDC-GR.

Cellular STR Loci and Genotyping Results				
Loci	STR Information of Cells		STR Information of Cells	
	Sample: LUAD-PDC		Sample: LUAD-PDC-GR	
	Allele1	Allele2	Allele1	Allele2
D5S818	11		11	
D13S317	8		8	
D7S820	10	11	10	11
D16S539	9		9	
VWA	17		17	
TH01	7		7	
AMEL	X		X	
TPOX	11		11	
CSF1PO	11		11	
D12S391	18		18	
FGA	23	25	23	25
D2S1338	19	20	19	20
D21S11	29	30	29	30
D18S51	15		15	
D8S1179	11	15	11	15
D3S1358	16		16	
D6S1043	13		13	
PENTAE	11	15	11	15
D19S433	13	15.2	13	15.2
PENTAD	9	13	9	13

**Table 3:** Cellular STR loci and genotyping results in LUAD-PDC and LUAD-PDC-OR.

Cellular STR Loci and Genotyping Results				
Loci	STR Information of Cells		STR Information of Cells	
	Sample: LUAD-PDC		Sample: LUAD-PDC-OR	
	Allele1	Allele2	Allele1	Allele2
D5S818	11		11	
D13S317	8		8	
D7S820	10	11	10	11
D16S539	9		9	
VWA	17		17	
TH01	7		7	
AMEL	X		X	
TPOX	11		11	
CSF1PO	11		11	
D12S391	18		18	
FGA	23	25	23	25
D2S1338	19	20	19	20
D21S11	29	30	29	30
D18S51	15		15	
D8S1179	11	15	11	15
D3S1358	16		16	
D6S1043	13		13	
PENTAE	11	15	11	15
D19S433	13	15.2	13	15.2
PENTAD	9	13	9	13

#### 2.3.2. Transcriptomic Profiling Uncovers Canonical Resistance Pathways in Patient-Derived Models

Transcriptome sequencing was performed on parental LUAD-PDCs and their Gefitinib- and Osimertinib-resistant derivatives to characterize molecular alterations underlying acquired resistance. Comparative transcriptomic analysis identified 1065 and 2103 differentially expressed genes (DEGs) in LUAD-PDC-GR and LUAD-PDC-OR sublines, respectively (Figure 3D,G), indicating substantial reprogramming.

Notably, volcano plots revealed significant dysregulation of established resistance genes. The Gefitinib-resistant model (Figure 3D) showed marked upregulation of *MET*, a gene frequently associated with resistance via amplification-induced bypass signaling and alternative pathway activation [19], and *AKT3*, a key effector in the PI3K-AKT survival pathway [11].

Similarly, the Osimertinib-resistant cells (Figure 3G) exhibited pronounced overexpression of *MET*, which is recognized drivers of resistance to third-generation EGFR-TKIs. Additionally, these cells showed upregulated expression of other resistance-associated receptors, including *PDGFRB* [20], which can activate alternative survival signaling through bypass mechanisms. The transcription factor *STAT5A*, which promotes cell survival and proliferation upon activation, was also elevated, contributing to the resistant phenotype [21,22]. These alterations collectively highlight the multifaceted mechanisms underlying acquired resistance to EGFR-targeted therapy.

To systematically understand the dysregulated pathways underlying resistance, KEGG pathway enrichment analysis was performed on the DEGs from both models. This analysis revealed significant enrichment of several canonical pathways with established roles in EGFR-TKI resistance mechanisms. Notably, both Gefitinib and Osimertinib resistant cells exhibited robust enrichment of the MAPK and PI3K-Akt signaling pathways (Figure 3E,H), two core pathways known to mediate resistance through maintenance of downstream proliferative and survival signals when EGFR is inhibited. The MAPK pathway facilitates bypass signaling through alternative activation of proliferative cascades, while the PI3K-Akt pathway provides critical anti-apoptotic signals that sustain cell survival despite EGFR-TKI treatment [11].

In addition to these shared mechanisms, Gefitinib resistant cells uniquely showed enrichment in ECM receptor interaction (Figure 3E), a pathway linked to cell adhesion-mediated drug protection and microenvironment interactions [23]. Osimertinib-resistant cells specifically exhibited enrichment of the JAK-STAT signaling pathway (Figure 3H), which has been increasingly associated with resistance to third-generation EGFR-TKIs through inflammatory signaling and immune modulation [24]. These results show that the biological pathways activated in this resistance models match those known to cause drug resistance in patients. This confirms that the patient-derived models accurately mimic the complex biology of clinical EGFR-TKI resistance.

To visualize expression patterns of genes within the enriched KEGG pathways, heatmaps were generated displaying the DEGs associated with key resistance pathways in both resistant models (Figure 3F,I). The majority of these genes showed marked upregulation in resistant sublines compared to parental cells, consistent with pathway activation. These results provide transcriptional evidence that critical resistance pathways are actively engaged in the patient-derived models.

Collectively, transcriptomic analyses demonstrate that patient-derived resistant models successfully recapitulate clinically relevant mechanisms of EGFR-TKI resistance. This work establishes a reliable platform for the subsequent discovery of potential resistance targets and exploration of underlying mechanisms.

### 2.4. Phenotypic and Molecular Characterization of EGFR-TKI-Resistant PC9 Models

**Figure 4:**
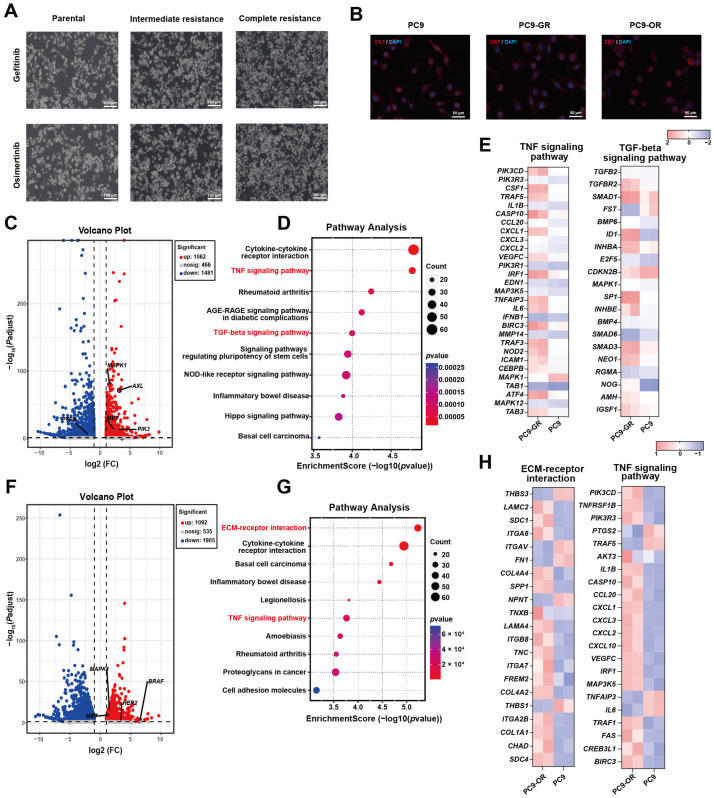
Phenotypic and molecular characterization of resistant PC9 models. (**A**) Representative images comparing the morphology of parental, intermediate, and complete resistant PC9 cells. (**B**) Immunofluorescence staining of CK7 in parental and resistant PC9 cells. (**C**–**E**) Transcriptomic analysis of Gefitinib-resistant model: (**C**) Volcano plot of differentially expressed genes in PC9-GR versus parental cells. (**D**) KEGG pathway enrichment analysis. (**E**) Heatmap displaying expression patterns of key genes from the pathways enriched in (**D**). (**F**–**H**) Transcriptomic analysis of Osimertinib-resistant model: (**F**) Volcano plot of differentially expressed genes in PC9-OR versus parental cells. (**G**) KEGG pathway enrichment analysis. (**H**) Heatmap of key gene expression from the pathways in (**G**).

Phenotypic and molecular characterization confirmed that the PC9-resistant models faithfully recapitulate established mechanisms of acquired resistance. Morphologically, both parental and resistant PC9 cells maintained a mixed population of round and spindle-shaped cells, indicating a stable cellular architecture throughout the induction process (Figure 4A). This preservation of lineage was further supported by immunofluorescence, which showed that the epithelial marker CK7 remained highly expressed in both PC9-GR and PC9-OR cells at levels comparable to parental controls (Figure 4B).

At the molecular level, transcriptomic profiling revealed the activation of canonical resistance pathways. In PC9-GR cells, volcano plot analysis identified significant upregulation of a cadre of established resistance-associated genes, including *PIK3CA* [25], *MAPK1* [26], *AXL* [27,28], and *MET* (Figure 4C). A similar pattern was observed in PC9-OR cells, with pronounced upregulation of *MAPK1*, *MET*, *HER2* [29], and *BRAF* [30] (Figure 4F).

KEGG pathway enrichment analysis demonstrated that the biological pathways activated in PC9 resistant models align with established mechanisms of EGFR-TKI resistance. In PC9-GR cells, significant pathway enrichment included TNF signaling, which has been documented to confer resistance through NF-κB activation [31], and TGF-beta signaling, a known driver of EMT-mediated resistance [32] (Figure 4D). Similarly, PC9-OR cells exhibited enrichment of ECM–receptor interaction, a pathway recognized for promoting resistance through integrin-mediated survival signaling [33], alongside TNF signaling activation (Figure 4G). Heatmap visualization of DEGs within enriched KEGG pathways demonstrated consistent upregulation of key resistance-associated genes in both PC9-GR and PC9-OR sublines compared to parental cells (Figure 4E,H). Collectively, these molecular signatures validate these models, confirming that they faithfully recapitulate well-established mechanisms of EGFR-TKI resistance.

### 2.5. Multi-Level Validation of MUC16 as a Potential EGFR-TKI Resistance Target

**Figure 5:**
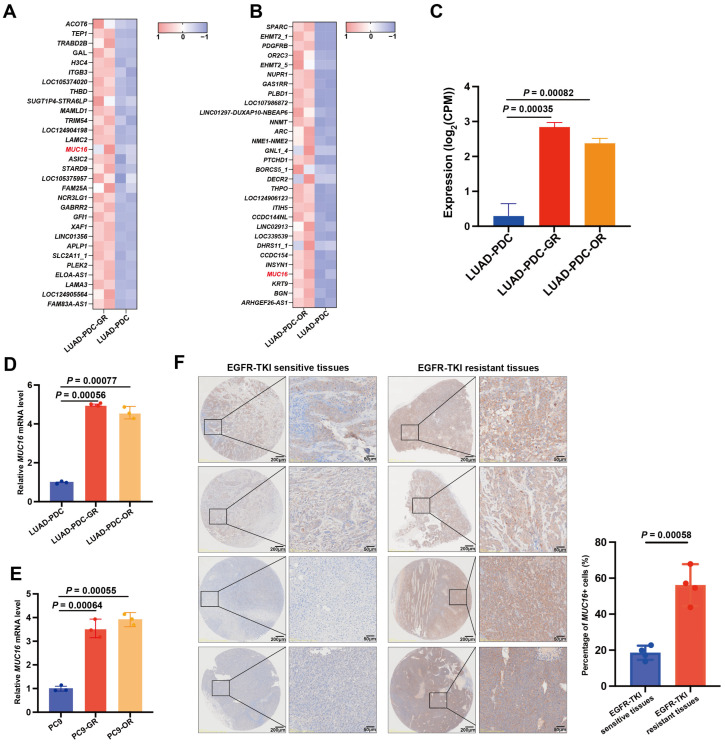
Cellular and clinical validation of MUC16 upregulation in EGFR-TKI resistance. (**A**,**B**) Heatmaps displaying the top 30 upregulated genes ranked by log_2_FoldChange value in (**A**) LUAD-PDC-GR and (**B**) LUAD-PDC-OR versus parental cells. *MUC16* is indicated among the top upregulated genes. (**C**) Normalized *MUC16* expression levels (log_2_(CPM)) in parental, GR, and OR cells from RNA sequencing data (*n* = 2). one-way ANOVA. Data presented as means ± SDs. (**D**) qPCR analysis of *MUC16* mRNA expression in LUAD-PDC-GR and LUAD-PDC-OR cells. (**E**) qPCR analysis of *MUC16* mRNA expression in PC9-GR and PC9-OR cells. (**F**) Immunohistochemical staining of *MUC16* in EGFR-TKI-resistant and sensitive LUAD tissues (*n* = 4 biological replicates). Two-sided unpaired Student’s *t*-test. Data presented as means ± SDs.

In addition to the most significantly altered transcripts, candidate genes were prioritized for functional validation based on the magnitude of differential expression and existing biological plausibility. *MUC16* emerged as a top candidate due to its consistent and marked upregulation across both resistant models (Figure 5A,B), coupled with established associations with lung cancer pathogenesis. Previous studies have reported that *MUC16* overexpression promotes lung cancer cell growth, invasion, and chemoresistance, potentially via suppression of p53 pathway genes. Moreover, *MUC16* mutations have been previously detected in genomic studies of EGFR-mutated NSCLC [34,35]. Quantification of the RNA-Seq data showed a substantial increase in *MUC16* gene transcript levels in both LUAD-PDC-GR and LUAD-PDC-OR cells compared to the parental line (Figure 5C).

The potential involvement of *MUC16* in EGFR-TKI resistance was assessed by examining its expression in established resistant cell models and clinical specimens. In patient-derived cells, *MUC16* expression was elevated in both LUAD-PDC-GR and LUAD-PDC-OR sublines compared to their parental counterparts (Figure 5D). Similarly, the EGFR-TKI-resistant PC9 models (PC9-GR and PC9-OR) demonstrated substantially higher *MUC16* levels than sensitive PC9 cells (Figure 5E). Clinical validation using immunohistochemistry on LUAD tissues revealed markedly stronger *MUC16* staining in EGFR-TKI-resistant tissues compared to treatment-sensitive controls (Figure 5F).

### 2.6. MUC16 Knockdown Reverses EGFR-TKI Resistance

**Figure 6:**
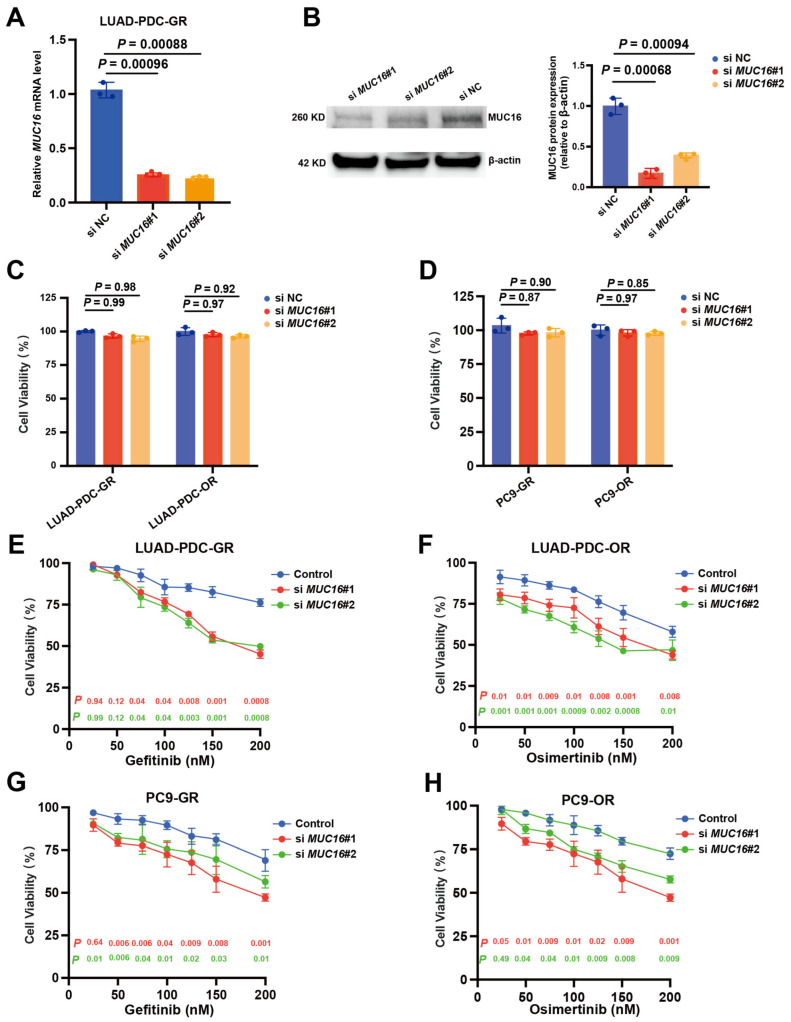
Functional validation of *MUC16* in mediating EGFR-TKI resistance. (**A**) Evaluation of siRNA-mediated knockdown efficiency of *MUC16* in LUAD-PDC-GR cells using two independent siRNAs (si*MUC16*#1 and si*MUC16*#2) and a non-targeting control siRNA. (**B**) Western blot analysis of *MUC16* protein levels following siRNA transfection in LUAD-PDC-GR cells. (**C**,**D**) Viability of (**C**) LUAD-PDC-GR, LUAD-PDC-OR, and (**D**) PC9-GR, PC9-OR cells following *MUC16* knockdown. (**E**–**H**) Dose–response curves of (**E**) LUAD-PDC-GR, (**F**) LUAD-PDC-OR (**G**) PC9-GR, and (**H**) PC9-OR cells transfected with control or *MUC16*-targeting siRNAs and treated with corresponding EGFR-TKIs (*n* = 3 technical replicates). Two-sided unpaired Student’s *t*-test. Data presented as means ± SDs.

To investigate the functional role of *MUC16* in maintaining the resistant phenotype, knockdown of *MUC16* was performed using two independent siRNAs, confirming knockdown at both mRNA (Figure 6A) and protein levels (Figure 6B). *MUC16* depletion alone did not significantly affect cell viability across all four resistant models—LUAD-PDC-GR, LUAD-PDC-OR, PC9-GR, and PC9-OR (Figure 6C,D), indicating that *MUC16* is dispensable for basal cell survival.

The effect of MUC16 knockdown on EGFR-TKI sensitivity was next examined. In LUAD-PDC-GR cells, sensitivity to Gefitinib was significantly enhanced by both si*MUC16*#1 and si*MUC16*#2 compared to controls (Figure 6E). Similarly, LUAD-PDC-OR cells showed increased sensitivity to Osimertinib following *MUC16* knockdown (Figure 6F). This re-sensitization effect extended to PC9-based models, with both PC9-GR (Figure 6G) and PC9-OR (Figure 6H) exhibiting restored drug sensitivity after *MUC16* silencing. These consistent results across models establish that *MUC16* contributes to EGFR-TKI resistance maintenance and that its targeted suppression can restore drug sensitivity without compromising basal cell growth.

### 2.7. MUC16 Is Overexpressed in LUAD and Associated with Poor Patient Prognosis

**Figure 7:**
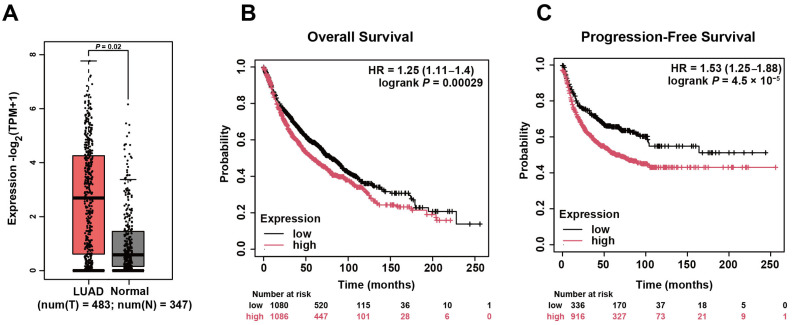
Clinical relevance of *MUC16* expression in LUAD. (**A**) Comparison of *MUC16* mRNA expression levels between normal lung and LUAD tissues from the GEPIA2 database. Wilcoxon rank-sum test. Data are presented as box plots. (**B**,**C**) Kaplan–Meier survival curves of LUAD patients from the Kaplan–Meier Plotter database stratified by low and high *MUC16* expression for overall survival (OS, left) and progression-free survival (PFS, right). Statistical significance was determined by log rank test.

The clinical relevance of *MUC16* was assessed by evaluating its expression and prognostic significance using public databases. *MUC16* mRNA levels were significantly higher in LUAD tissues than in normal lung samples (Figure 7A). Survival analyses further revealed that elevated *MUC16* expression correlated strongly with poorer overall survival (OS; HR = 1.25, logrank *p* = 0.00029) and reduced progression-free survival (FP; HR = 1.53, logrank *p* = 4.5 × 10^−5^) in LUAD patients (Figure 7B,C), highlighting its role in aggressive disease progression.

Combined with the functional evidence that *MUC16* knockdown restores EGFR-TKI sensitivity, these clinical findings establish *MUC16* as both a mechanistic contributor to resistance and a prognostically relevant biomarker. The identification and validation of *MUC16* were enabled by these EGFR-TKIs resistant models, demonstrating the utility of this platform for discovering clinically significant targets that may be missed in conventional systems.

## 3. Discussion

Acquired resistance to EGFR-TKIs continues to pose a major clinical challenge in EGFR-mutant LUAD. In this study, we established and characterized in vitro models of EGFR-TKI resistance that recapitulate resistance to both first- and third-generation inhibitors. Transcriptomic profiling of these models identified *MUC16* as a candidate mediator of resistance, and functional studies confirmed that *MUC16* silencing resensitizes resistant cells to EGFR-TKIs. Clinical correlation further revealed an association between *MUC16* expression and adverse patient outcomes, underscoring its potential clinical relevance and validating the utility of our model system for resistance mechanism discovery.

A key strength of this study lies in the establishment of a comprehensive resistance platform that integrates the reproducibility of conventional cell lines with the clinical relevance of patient-derived cultures. By developing resistant variants in parallel from both PC9 cells and PDCs, we created a complementary system that leverages the unique advantages of each model. This approach provides a robust tool for dissecting the mechanisms of acquired resistance.

The role of *MUC16* in promoting tumorigenesis and inducing drug resistance has been documented in various cancers. In lung cancer, *MUC16* was reported to promote tumor growth and confer chemoresistance by suppressing p53 via the JAK2/STAT3/GR/TSPYL5 axis [34]. Furthermore, *MUC16*-mediated inhibition of immune synapse formation and protection of ovarian cancer cells from NK cell cytotoxicity raises the possibility of non-cell-autonomous mechanisms in resistance [36]. Although our study firmly establishes *MUC16* as a contributor to EGFR-TKI resistance, its precise molecular mechanisms in LUAD warrant further investigation.

Beyond its mechanistic involvement in EGFR-TKI resistance, *MUC16* holds significant clinical potential in EGFR-mutant LUAD. Elevated expression of *MUC16* has been consistently linked to tumor progression, aggressive cellular phenotypes, adverse clinical outcomes, and therapy resistance across multiple cancer types, including NSCLC, ovarian, bladder, and gastric cancers [37,38,39,40]. The persistent upregulation of *MUC16* observed in the EGFR-TKI-resistant LUAD models underscores its potential utility as a biomarker for acquired resistance. Moreover, emerging clinical evidence suggests that *MUC16* may have predictive value for EGFR-TKI therapy response. A multicenter retrospective study found that elevated baseline serum CA125 levels, a soluble form of MUC16, was an independent predictor of shorter progression-free survival in LUAD patients undergoing EGFR-TKI treatment [41].

Given its clinical significance, *MUC16* has also been investigated as a therapeutic target in various solid tumors using antibody-drug conjugates and immune-based strategies [42,43]. *MUC16*-targeting antibodies have been utilized as theranostic agents, facilitating both imaging and targeted therapy in *MUC16*-expressing cancers [44]. Our findings offer a robust preclinical justification for extending *MUC16*-targeted strategies to EGFR-TKI-resistant LUAD, particularly in combination with EGFR-TKIs to overcome resistance. Importantly, MUC16 status can be assessed through clinically feasible methods, such as immunohistochemistry of biopsy samples or monitoring soluble CA125 levels in blood [45]. This positions *MUC16* as a highly tractable target for translational research and biomarker-guided combination therapies in EGFR-mutant LUAD.

Future work will focus on elucidating the molecular pathways through which MUC16 promotes resistance, including the identification of its binding partners and downstream signaling effectors. Validation of *MUC16* function in vivo using patient-derived xenograft models with genetic or pharmacologic *MUC16* inhibition will be critical to establish its role in a more physiologically relevant context. Furthermore, evaluating MUC16-targeted strategies using pharmacological or RNAi approaches, alone or in combination with EGFR-TKIs, will be essential to assess their potential to resensitize resistant tumors and accelerate clinical translation.

In conclusion, this study establishes a robust platform for dissecting EGFR-TKI resistance by integrating both conventional and patient-derived models. Leveraging this system, we identified and functionally validated *MUC16* as a mediator of resistance, positioning it as a promising therapeutic target. While further validation in vivo and in additional patient-derived systems is the critical next step, our findings provide a strong preclinical foundation for developing potential strategies to overcome EGFR-TKI resistance in LUAD.

## 4. Materials and Methods

### 4.1. Cell Culture and Reagents

LUAD patient-derived cancer cells (LUAD-PDCs) and PC9 cells were cultured in Dulbecco’s Modified Eagle Medium (DMEM) supplemented with 10% fetal bovine serum (ThermoFisher, Scientific, Waltham, MA, USA) and 1% penicillin/streptomycin. Cells were maintained as an adherent monolayer in sterile culture dishes at 37 °C in a humidified atmosphere containing 5% CO_2_. When reaching 80–90% confluence, the cells were passaged using trypsin. Detached cells were collected into a 15 mL centrifuge tube and centrifuged at 1000 rpm for 5 min. After centrifugation, the supernatant was discarded, and the cell pellet was resuspended in fresh complete medium for subsequent experiments. Primary LUAD cells were isolated from tumor tissues obtained from patients with LUAD at West China Hospital of Sichuan University, with ethical approval number 2022-1471. Gefitinib (S1005, Selleck Chemicals, Houston, TX, USA) and Osimertinib (S1450, Selleck Chemicals, Houston, TX, USA) were dissolved in dimethyl sulfoxide (DMSO) to prepare stock solutions, which were stored at −20 °C until use.

### 4.2. Isolation and Culture of Lung Adenocarcinoma Patient-Derived Cells

Fresh LUAD tissues obtained from surgically resected individual donors were promptly processed in cold, antibiotic-containing preservation medium within 1 h. Briefly, after rinsing with DPBS to remove blood clots, the tissues were meticulously minced into approximately 1 mm^3^ fragments using sterile surgical blades. The fragments were then subjected to enzymatic digestion in a solution of 0.5 mg/mL collagenase IV (17104-019, Gibco, Waltham, MA, USA) and 1 mg/mL collagenase I (17100-017, Gibco, Waltham, MA, USA) dissolved in DMEM base medium. The digestion was carried out on an orbital shaker set at 180 rpm at 37 °C for 40 min, with manual pipette mixing (8–10 aspirations) performed every 10 min to facilitate tissue dissociation. The resulting cell suspension was filtered through a 100 μm cell strainer (25418675, Biosharp, Hefei, China) to remove undigested debris. Red blood cells were subsequently lysed by a brief incubation with ACK lysis buffer (GA2502021, Biosharp, Hefei, China). The isolated cells were washed twice with DPBS and resuspended in complete growth medium (DMEM supplemented with 10% heat-inactivated FBS, 1% Penicillin-Streptomycin). The cells were maintained at 37 °C in a humidified 5% CO_2_ incubator. The medium was refreshed every 2–3 days, and cells were passaged upon reaching 80–90% confluence.

### 4.3. Establishment of EGFR-TKI-Resistant Models

To establish EGFR-TKI-resistant cells, Gefitinib-resistant and Osimertinib-resistant variants were derived from PC9 and LUAD-PDCs parental cells via continuous exposure to increasing drug concentrations. Resistance was induced using a stepwise escalation protocol, starting from low initial concentrations (for PC9 cells, treatment began at 10 nM Gefitinib or 30 nM Osimertinib, while for LUAD-PDCs, initial concentrations were 5 nM Gefitinib or 4 nM Osimertinib) with increments of 1.5-fold. Cells were treated at each concentration for 48 h, followed by a drug-free recovery period to allow surviving cells to proliferate. This cycle was repeated with increasing TKI concentrations, enabling the selection of resistant populations. Over approximately 90 days, drug concentrations were gradually increased until reaching the final maintenance doses of 1 μM for Gefitinib and 500 nM for Osimertinib. Cells were then maintained at these concentrations for an additional 60 days to achieve stable resistance. After a total of about 180 days of selection, all resistant cell models demonstrated sustained proliferation under respective EGFR-TKI treatments and retained resistance phenotypes during subsequent passaging.

### 4.4. Cell Viability Assays

Cell viability was assessed using the CCK-8 assay kit (DOJINDO, Kumamoto, Japan) according to the manufacturer’s instructions. Briefly, cells were digested, counted, and seeded into 96-well plates at a density of 3000 cells per well in 100 μL DMEM medium, with 3 replicate wells per condition. After 24 h of incubation, cells were treated with various drug concentrations for 48 h. The medium was then replaced with 100 μL of fresh DMEM medium without serum or antibiotics, containing 10% CCK-8 reagent. Plates were further incubated for 2 h until an orange-red color developed. Absorbance was measured at 450 nm using a microplate reader (Bio-Tek, Winooski, VT, USA).

### 4.5. RNA-Seq

For transcriptome sequencing, total RNA was extracted and subjected to rigorous quality control. mRNA was enriched using poly-T magnetic beads, fragmented, and converted into cDNA. After end repair, adenylation, and adapter ligation, libraries were constructed and selected for fragments of 370–420 bp. Quality assessment was performed using Qubit and Agilent 2100. Qualified libraries were sequenced on the Illumina NovaSeq 6000 platform to generate 150 bp paired end reads. Bioinformatics analysis included quality control with Q20/Q30 filtering, alignment to the reference genome using HISAT2, gene expression quantification via featureCounts and FPKM, and differential expression analysis with DESeq2 or edgeR. DEGs were identified with |log_2_FC| ≥ 1 and *p* < 0.05 criteria, followed by GO and KEGG enrichment analyses.

### 4.6. Quantitative PCR

Total RNA was reverse transcribed into cDNA for gene expression analysis. qPCR was performed using a SYBR Green-based kit (Novozan, Nanjing, China). Each 10 μL reaction contained 5 μL SYBR Green mix, 0.5 μL forward primer, 0.5 μL reverse primer (Tsingke Biotechnology, Beijing, China), and 4 μL cDNA template. The following primers were used for *MUC16* amplification:

Forward: CCAGTCCTACATCTTCGGTTGT
Reverse: AGGGTAGTTCCTAGAGGGAGTT

All samples were run in technical triplicates. The amplification protocol consisted of an initial denaturation at 95 °C for 30 s, followed by 40 cycles of 95 °C for 10 s and 60 °C for 30 s. Gene expression levels were normalized to GAPDH and calculated using the 2^−ΔΔCT^ method. Biological replicates were included for all experiments.

### 4.7. siRNA Transfection

For siRNA transfection, cells were seeded in 96-well or 6-well plates at densities of 5000 or 4 × 10^5^ cells per well, respectively, and cultured overnight. Transfection complexes were prepared by mixing siRNA (20 μM) with jetBUFFER, followed by brief vortexing and addition of jetPRIMER (Polyplus, Ilkirch, France). After 10 min of incubation at room temperature, the mixture was diluted with complete medium to achieve a final siRNA concentration of 20 nmol/L. The complexes were added to the cells at 100 μL/well (96-well plate) or 2 mL/well (6-well plate). Transfection efficiency was evaluated using qPCR after 48 h. For drug efficacy assays, compounds were added 24 h post-transfection and incubated for an additional 48 h before analysis. Two specific siRNAs (Tsingke Biotechnology, Beijing, China) were used:

siRNA#1: sense strand: CAACUAUGGAUGUCACUAA, antisense strand: UUAGUGACAUCCAUAGUUG.

siRNA#2: sense strand: GAUGCAACAUUCAUACCAA, antisense strand: UUGGUAUGAAUGUUGCAUC.

### 4.8. Animal Studies

BALB/c nude male mice (18–20 g, 4–6 weeks old) were supplied by Beijing Vital River Laboratory Animal Technology Co., Ltd. (Beijing, China). and housed under SPF conditions (24 ± 2 °C, 55 ± 5% humidity, 12-h light/dark cycle) with free access to food and water. Primary LUAD cells were isolated from tumor tissues obtained from patients with LUAD at West China Hospital of Sichuan University, with ethical approval (IRB Approval No. 2022-1471, approved 20 September 2022). Cells (5 × 10^6^ per mouse) mixed with Matrigel were inoculated subcutaneously into the right flank. When tumor volumes reached approximately 100 mm^3^, mice were randomly assigned using a computer-generated sequence to three groups (*n* = 4): vehicle, Gefitinib (50 mg/kg, oral gavage), or Osimertinib (15 mg/kg, oral gavage). Treatments continued for 20 days. No animals were excluded from analysis. Blinding was not employed during the experiment. Tumors were excised, photographed, and weighed at the endpoint. All procedures were approved by the Institutional Animal Care and Use Committee of West China Hospital, Sichuan University (IACUC Approval No. 20241025009, approved 25 October 2024). Humane endpoints were set as tumor volume > 2000 mm^3^ or severe distress; no adverse events occurred. Data are presented as mean ± SD and analyzed using GraphPad Prism 9.0 with one-way ANOVA and Tukey’s test.

### 4.9. Western Blot Analysis

Total protein was extracted from cultured cells using RIPA lysis buffer supplemented with protease and phosphatase inhibitors. Protein concentration was determined using a BCA protein assay kit (P0010, Beyotime, Shanghai, China). Equal amounts of protein (15 μg per lane) were separated by SDS-PAGE using precast gels (M00662, Genscript, Nanjing, China) and subsequently transferred onto PVDF membranes. After blocking with 5% non-fat milk in TBST for 1 h at room temperature, the membranes were incubated overnight at 4 °C with primary antibody against *MUC16* (610920, Selleck Chemicals, Houston, TX, USA, https://www.selleckchem.com/ (accessed on 15 August 2025)) at appropriate dilution. Following three washes with TBST, the membranes were incubated with HRP-conjugated secondary antibody (AS014, Abclonal, Wuhan, China) for 1 h at room temperature. Protein bands were visualized using enhanced chemiluminescence substrate and imaged with a chemiluminescence detection system. β-actin (Beyotime, Cat# AT819) was used as the loading control for normalization.

### 4.10. Sanger Sequencing

Genomic DNA was extracted from early-passage LUAD-PDC cells using a commercial kit. The EGFR exon 19 region was amplified by PCR with specific primers (Forward: 5′-GCAATATCAGCCTTAGGTGC-3′; Forward: 5′-TGAGCAGGGTCTAGAGCAGA-3′). The PCR products were purified and sequenced by Sanger sequencing. Sequence chromatograms were aligned against the wild-type EGFR reference sequence (NG_007726.3).

### 4.11. Immunohistochemistry

IHC was performed on formalin-fixed, paraffin-embedded lung adenocarcinoma tissue sections. Briefly, sections were deparaffinized in xylene and rehydrated through a graded ethanol series. Antigen retrieval was carried out using citrate buffer (pH 6.0) in a microwave heating system. Endogenous peroxidase activity was blocked with 3% hydrogen peroxide. After blocking, sections were incubated overnight at 4 °C with primary antibody against *MUC16* (Selleck, Cat# 610920) at appropriate dilution. Following washes, sections were incubated with HRP-conjugated secondary antibody (Abclonal, Cat# AS014) for 30 min at room temperature. Diaminobenzidine (DAB) was used as the chromogen, and hematoxylin was applied for counterstaining. Finally, sections were dehydrated, cleared, and mounted for microscopic examination.

### 4.12. Immunofluorescence

For cytokeratin 7 (CK7) immunofluorescence staining, cells were seeded on coverslips in 24-well plates and cultured until reaching 70–80% confluence. Cells were then fixed with 4% paraformaldehyde for 15 min at room temperature, permeabilized with 0.1% Triton X-100 (Beyotime, China, ST795) for 10 min, and blocked with 5% bovine serum albumin (BSA, Beyotime, China, ST023) for 1 h. Subsequently, cells were incubated with a primary antibody against CK7 (ab181598, Abcam, Cambridge, UK) overnight at 4 °C. After washing with PBS, cells were incubated with a CY5-conjugated goat anti-rabbit secondary antibody (CSB-MA566015, Bioss, Beijing, China) for 1 h at room temperature in the dark. Nuclei were counterstained with DAPI (1 µg/mL) for 5 min. Coverslips were mounted onto glass slides with antifade mounting medium, and images were captured using a fluorescence microscope.

### 4.13. STR Profiling

Cell authentication was performed through STR analysis. Genomic DNA from parental and resistant cells was amplified using the PowerPlex 16 HS System, which examines 15 STR loci plus amelogenin. Capillary electrophoresis results confirmed >95% profile match between sensitive and resistant cells, verifying their common genetic origin and absence of cross-contamination during resistance induction.

### 4.14. Statistical Analysis

Data are presented as mean ± standard deviation from at least three independent experiments. Statistical differences between multiple groups were analyzed using one-way analysis of variance (ANOVA), followed by Tukey’s honestly significant difference (HSD) post hoc test for pairwise comparisons. For comparisons between two groups, either the independent samples *t*-test (for data meeting assumptions of normality and homogeneity of variances) or the Mann–Whitney U test (for non-normally distributed data) was applied. All statistical analyses were performed using SPSS (version 20.0, IBM, Armonk, NY, USA), with a *p*-value ≤ 0.05 considered statistically significant.

## Data Availability

The original data presented in the study are openly available in GEPIA2 database and the Kaplan-Meier Plotter database at https://doi.org/10.1093/nar/gkz430 and https://doi.org/10.1371/journal.pone.0082241.

## Abbreviations

The following abbreviations are used in this manuscript:

EGFR	Epidermal Growth Factor Receptor
TKI	Tyrosine Kinase Inhibitor
LUAD	Lung Adenocarcinoma
PDC	Patient-Derived Cell
EMT	Epithelial-to-Mesenchymal Transition
